# Editorial: The individualization of antiplatelet therapy in coronary artery disease: escalation or de-escalations

**DOI:** 10.3389/fcvm.2023.1219689

**Published:** 2023-06-06

**Authors:** Sang Yeub Lee, Tobias Geisler, Zuzana Motovska, Young-Hoon Jeong

**Affiliations:** ^1^ CAU Thrombosis and Biomarker Center, Chung-Ang University Gwangmyeong Hospital, Gwangmyeong, Republic of Korea; ^2^ Department of Internal Medicine, Chung-Ang University College of Medicine, Seoul, Republic of Korea; ^3^ Department of Cardiology and Angiology, University Hospital Tübingen, Eberhard Karls Universtität Tübingen, Tübingen, Germany; ^4^ Cardiocenter, Third Faculty of Medicine, Charles University and University Hospital Kralovske Vinohrady, Prague, Czech Republic

**Keywords:** individualization, antiplatelet therapy, coronary artery disease, colchicine, de-escalation, ethnicity

**Editorial on the Research Topic** The individualization of antiplatelet therapy in coronary artery disease: escalation or de-escalations

Among patients with acute coronary syndrome (ACS) undergoing percutaneous coronary intervention (PCI), the use of potent P2Y_12_ inhibitor (e.g., prasugrel or ticagrelor) is associated with a reduction in ischemic events as well as an increase in bleeding events compared with clopidogrel treatment. Therefore, finding an optimal balance between the efficacy and safety of antiplatelet strategies after PCI (i.e., optimal disease entity, regimen, potency, and duration) has been one of the challenging projects. Recently, the main issue in this field has shifted toward reducing serious bleeding without increasing ischemic risk. Thereafter, numerous clinical trials have evaluated clinical benefits of the de-escalation antiplatelet strategies compared with the conventional strategy (1, 2).

This Research Topic section contains five papers and aims at widening the current knowledge on the personalized antithrombotic therapy, mostly targeting on “de-escalation strategy” of dual antiplatelet therapy (DAPT) in PCI-treated patients with coronary artery disease (CAD).

## De-escalation strategy of antiplatelet therapy in acute coronary syndrome: reviews

Two articles reviewed the recent interest in the development of antiplatelet regimens aimed at reducing bleeding without any trade-off in ischemic events. Mattia Galli et al. and Mohamed Farag et al. reviewed the rationale, regimens, timing and combined limitations of “de-escalation antiplatelet strategy” in patients with ACS. **De-escalation of DAPT duration** appears favorable, with reduction in bleeding, mostly without increase in ischemic events, despite an increase in ischemic events in some studies using abbreviated DAPT. Likewise, **de-escalation of DAPT intensity** appears to signiﬁcantly reduce major bleeding, without signiﬁcant effect on ischemic events. Timing of de-escalation strategy may not be fixed because this strategy has been tested at different time points: (1) 2–3 days post-PCI (e.g., guided de-escalation); (2) after 1–3 months of DAPT, followed by P2Y_12_ inhibitor monotherapy (e.g., clopidogrel or ticagrelor); and (3) after 3–6 months of DAPT, followed by aspirin monotherapy. Since the clinical benefit of de-escalation strategy may be influenced by a number of factors (e.g., ethnicity, gender, and disease entity and phase), this concept in the current era can be applicable mostly for selected clinical scenarios, such as high-bleeding risk (HBR) (3).

Recent meta-analyses have compared the net benefits of various de-escalation strategies in ACS patients (4, 5). Compared with the conventional DAPT strategy, most of the current de-escalation strategies have shown the reduction of clinical events, except of increased risk in ischemic event during short-term DAPT, followed by aspirin monotherapy. Intensity de-escalation of P2Y_12_ inhibitor showed the best benefit in reducing the net clinical event, while short-term DAPT followed by antiplatelet monotherapy was most beneficial in decreasing major bleeding. Meanwhile, a recent individual patient meta-analysis demonstrated that the de-escalation strategy significantly reduced the risks of ischemic (hazard ratio [HR], 0.761; 95% confidence interval [CI], 0.597–0.972; log rank *P* = 0.029) and bleeding (HR, 0.701; 95% CI, 0.606–0.811; log rank *P* < 0.001) endpoints together (5). Compared to the guided de-escalation, the unguided de-escalation had a significantly larger impact on reducing bleeding endpoint (*P* for interaction = 0.007).

## De-escalation strategy of antiplatelet therapy in East Asian patients

East Asian patients have been shown to be more vulnerable to bleeding and less thrombogenic to ischemic event compared with Caucasian patients, a condition known as the “East Asian paradox” (6, 7). In addition, responsiveness to potent P2Y_12_ inhibitor appears greater in East Asians than Westerners. Therefore, clinical benefit of de-escalation strategy in ACS patients may be pronounced in these patients. A recent meta-analysis indicated the unique risk–benefit trade-off of de-escalation strategy in East Asian vs. Caucasian patients (8). In East Asian patients, reduction of DAPT intensity or duration could minimize bleeding without safety concerns. In Caucasian patients, reduction of DAPT intensity may incur an ischemic penalty, while DAPT abbreviation has no overall benefit.

Min-Gyu Kang et al. demonstrated potent antiplatelet effect of standard-dose ticagrelor (i.e., 90-mg twice daily), and its associations with short-term adverse events and DAPT discontinuation. First, reversibly-binding ticagrelor showed the different cutoff for 1-month bleeding episodes compared with irreversibly-binding clopidogrel [≤20 vs. ≤110 P2Y12 reaction unit (PRU) measured by VerifyNow assay]. Second, patients on ticagrelor showed the higher risks of 1-month bleeding compared with those on clopidogrel [any bleeding: 45.6% vs. 23.6%; odds ratio (OR), 2.71]. Third, early occurrence of bleeding episode was significantly associated with low level of PRU (OR, 2.68). Fourth, type of P2Y_12_ inhibitor (ticagrelor vs. clopidogrel: OR, 2.19) and bleeding episode (OR, 2.94) were independent predictors for dyspnea occurrence. During standard-dose ticagrelor in East Asian patients, prevalence of low platelet reactivity was very popular (PRU ≤ 20: 68.1%) compared with that (PRU ≤ 110: 16.5%) during 75-mg clopidogrel, which may suggest the unmet need to develop up-front de-escalation strategy with reduced-dose ticagrelor in these patients.

## Personalized allocation of aspirin for secondary prevention of coronary artery disease

Recent clinical trials have focused early discontinuation of aspirin following PCI. However, aspirin resistance determined by platelet function test may be associated with an increased risk of thrombotic event, prominent during the early phase post-PCI. In a large-scale PCI cohort (*n* = 7,090), aspirin resistance (the highest quintile measured by Multiplate assay) showed a significantly higher risk of death or stent thrombosis at 1 year (OR, 1.78; 95% CI, 1.39–2.27; *P* < 0.0001) (9). Therefore, adequate suppression of thromboxane synthesis is required to prevent occurrence of thrombotic events in high-risk patients with ACS. The responsiveness to aspirin in such patients could be improved by twice-daily aspirin administration. Nischal N Hegde et al. suggested the laboratory criteria to identify subjects who would benefit from a twice-daily aspirin dose. Twice-daily aspirin was effective if serum thromboxane-B2 levels at 4 h are <3,100 and >3,100 pg/ml at 24 h. If thromboxane-B2 at 4 h and 24-hours is >3,100 pg/ml, a twice-daily aspirin did not suggest enough antiplatelet effect and switching with potent P2Y_12_ inhibitors may be another option overcoming this huddle. Because low-dose aspirin could not achieve adequate inhibition of thromboxane-B2 following PCI in high-risk patients, alternative strategy against this issue would be required in these subjects.

## Collagen-induced platelet activation as a potential target of colchicine

Colchicine with anti-inflammatory effect demonstrates the clinical benefit against ischemic events in patients with stable angina or ACS (10, 11). Gabrielle J. Pennings et al. focused on antiplatelet effect of colchicine, primarily targeting on collagen-induced platelet activation via glycoprotein (GP)VI. Concentration of therapeutic-dose colchicine led to a significant decrease in collagen-induced platelet aggregation and altered GPVI level. Switching from aspirin to colchicine could be another plausible de-escalation strategy in ACS patients with enhanced inflammation, which effect should be tested in the future trials.

## Summary

The present Research Topic indicates an overview of the need and possibilities of individualizing antiplatelet therapy based on the current knowledge and available evidence (Table 1). Although de-escalation antiplatelet strategy represents a very promising concept, its globalization still has a number of limitations in ACS patients with high-ischemic profiles. Application of de-escalation DAPT strategy (e.g., reduced-dose prasugrel or ticagrelor) may be a default strategy option in East Asian patients with HBR phenotype. Furthermore, early discontinuation of aspirin may not be applicable for all-comers following PCI and its switching with colchicine is a potential treatment that could be available in the future.

**Table 1:** Information and highlights of the articles in the research topic.

Authors	Key challenges in the field	Objectives of the study	Highlights of the study
Mattia Galli et al. (Review article)	There has been a great interest in the development of antiplatelet regimens aimed at reducing bleeding without any trade-off in ischemic events.	To elucidate the rationale, appraise the evidence and provide practical recommendations on the use of a de-escalation strategy of antiplatelet therapy in patients with ACS.	A de-escalation strategy of antiplatelet therapy represents a very promising strategy for reducing bleeding without any trade-off in ischemic events in patients with ACS. The use of a de-escalation in lieu of a standard 12-months DAPT with potent P2Y_12_ inhibitors should always be considered after a careful assessment of individual bleeding and ischemic risks, and of individual response to an antiplatelet agent.
Mohamed Farag et al. (Mini-review)	The trials which have evaluated the safety and efficacy of de-escalation of DAPT can be broadly divided into studies evaluating a shorter duration of DAPT, and those studies in which DAPT that includes a potent P2Y_12_ inhibitor is compared to less intense DAPT, mainly clopidogrel or reduced-dose prasugrel.	To review strategy of de-escalation of DAPT duration or intensity in ACS patients undergoing PCI and summarize the limitations of studies to date, gaps in evidence and make recommendations for future studies.	De-escalation of DAPT duration post-ACS to monotherapy appears favorable, with reduction in bleeding, mostly without increase in MACE, although an increase in ischemic events was noted in some studies with abbreviated DAPT. Likewise, de-escalation of DAPT intensity appears to signiﬁcantly reduce major bleeding, without signiﬁcant effect on MACE.
Min-Gyu Kang et al. (Original article)	Clinical evidence raises the issues regarding the high risk of adverse events and serious bleeding in East Asian patients receiving standard-dose ticagrelor treatment.	To evaluate the association between adverse events and their associations with premature discontinuation of DAPT.	This study shows the different cutoff of low platelet reactivity during the reversible (ticagrelor) vs. irreversible P2Y_12_ inhibitor (clopidogrel). Early occurrence of bleeding and dyspnea is very common during standard-dose ticagrelor treatment in East Asian patients, which show a close association with premature DAPT discontinuation.
Nischal N Hegde et al. (Original article)	Some patients treated with once-daily aspirin may have an incomplete 24-h suppression of thromboxane-B2 synthesis due to increased platelet turnover. The response could be improved in such patients by twice-daily aspirin administration.	To identify such a group of patients who would beneﬁt from a twice-daily dose of aspirin.	Twice-daily aspirin may be beneﬁcial if serum thromboxane-B2 levels at 4 h are < 3,100 and > 3,100 pg/ml at 24 h. If thromboxane-B2 levels at 4 and 24 h is < 3,100 pg/ml but if there is a > 10% rise in serum thromboxane B2 at 24 h as compared to that at 4 h, then twice-daily aspirin should be considered.
Gabrielle J. Pennings et al. (Perspectives)	Colchicine has been demonstrated to reduce cardiac events in people with coronary artery disease. Recent studies have identified that colchicine may work as both an anti-inflammatory and antiplatelet agent.	To highlight the need to evaluate collagen-mediated platelet activation in clinical environments and clarify the potential role of this under-investigated activation pathway in mediating the effects of colchicine as an adjunctive therapeutic agent	Colchicine and other emerging therapies may modulate platelet function in ways undetected by conventional platelet function assays. Diversifying our approach to platelet activation *in vivo*, and considering the effect of agents such as colchicine on platelets and not only inflammatory pathways may reveal new clinical opportunities for patient care without the associated bleeding risk.

ACS, acute coronary syndrome; DAPT, dual antiplatelet therapy; MACE¸ major adverse cardiovascular event.
